# Learning curve analysis of robotic-assisted total knee arthroplasty with a Chinese surgical system

**DOI:** 10.1186/s13018-023-04382-4

**Published:** 2023-11-27

**Authors:** Haoran Zhang, Xizhuang Bai, Huisheng Wang, Zhiyong Zhu, Xi Li

**Affiliations:** Department of Sports Medicine and Joint Surgery, The People’s Hospital of Liaoning Province, The People’s Hospital of China Medical University, 33 Wenyi Road, Shenyang, 110000 China

**Keywords:** Accuracy, Learning curve, Operative time, Robot-assisted surgery, Total knee arthroplasty

## Abstract

**Purpose:**

The aim of this study was to analyze the learning curve of total operative time, bone cutting accuracy, and limb alignment in total knee arthroplasty (TKA) using a Chinese image-based knee surgery robot known as HURWA. Additionally, a comparison was conducted with conventional TKA to ascertain the benefits of robotic-assisted TKA.

**Methods:**

In this retrospective study, we analyzed a series of patients (*n* = 90) who underwent robotic-assisted total knee arthroplasty using the HURWA robot between December 2021 and October 2022. The procedures were performed by one of three orthopedic surgeons with varying levels of experience. As a control group, we selected the last 30 conventional TKA cases performed by each of these three surgeons. To determine the learning curve, we recorded the operative time, bone cutting error, and pre- and post-surgery radiographs.

**Results:**

The study found no significant differences in total operative time, bone cutting accuracy, or limb alignment among the three surgeons. Of the three surgeons, surgeon 1, who had the most experience in joint arthroplasty, reached the learning curve in case 8, with the shortest bone cutting time and robot time. Surgeon 2 reached the learning curve in case 16, while surgeon 3 reached the learning curve in case 9. There was no observable learning curve effect for bone cutting accuracy and limb alignment. However, the percentage of cases where limb alignment differed from preoperative planning by 3° or less was higher in robotic-assisted TKA (77.97%) than in conventional TKA (47.19%).

**Conclusion:**

The study determined that the learning curve for robotic-assisted TKA using the HURWA knee surgery robot ranged from 8 to 20 cases. No observable learning curve effect was detected for bone cutting accuracy or limb alignment. Experienced surgeons using the HURWA robot for bone cutting took less time and reached the learning curve earlier. The HURWA robot achieved better limb alignment without depending on the experience of conventional TKA.

## Introduction

Knee osteoarthritis (KOA) is a joint disease characterized by degenerative cartilage and osteophytes, which can result in painful deformities and limited mobility in the knee joint [1]. Currently, approximately 37 million individuals in China suffer from knee osteoarthritis [2]. Total knee arthroplasty (TKA) is the most effective treatment for end-stage KOA, and the number of Conventional-TKA (C-TKA) surgeries in the country has increased by 5.9 times in the last decade, with over 375,000 cases performed in 2019 alone [3].

In recent years, the development of robot-assisted TKA (RA-TKA) has enabled surgeons to more easily plan and adjust limb alignment as well as joint line obliquity (JLO). Preoperative CT scans or intraoperative bone morphology capture allow the surgeon to plan the implant position and bone cutting volume, while a robotic arm-controlled grinding drill or bone cutting guide enables precise bone cutting operations to achieve the MA or KA concept [4, 5]. Kayani et al. [6] found that experienced surgical surgeons reached proficiency after performing seven RA-TKA procedures. Vermue et al. [7] concluded that experienced surgeons are more likely to reach the learning curve first, and that there is no difference in gap balance and limb alignment with other surgeons after proficiency. These findings have contributed to the increasing popularity of RA-TKA.

China has recently launched the HURWA (HURWA-ROBOT Technology Co. Ltd, Beijing, China) knee surgery robot, an image-based, semiautomatic robotic arm bone cutting knee surgery robot [8]. The HURWA is an open platform that is compatible with various brands of knee implants. Zheng et al. [9] reported superior limb alignment and postoperative scores following HURWA RA-TKA compared to C-TKA.

Based on the current research focus on RA-TKA, we can pose five questions and three assumptions about the HURWA system.

Questions:

1. Can the HURWA system decrease the operative time for RA-TKA compared to conventional TKA?
2. Do surgeons with different levels of experience in C-TKA have consistent operative times when using the HURWA system for RA-TKA?
3. What is the learning curve for operative time when using the HURWA system for RA-TKA, and how does it differ between surgeons with varying levels of experience?
4. Is the bone cutting accuracy of the HURWA system affected by the surgeon's experience in C-TKA?
5. Is the postoperative limb alignment accuracy of the HURWA system affected by the surgeon's experience in C-TKA?

Assumptions:

1. It is likely that HURWA RA-TKA will take longer than C-TKA, but surgeons with different levels of experience will become proficient in less than 20 cases, and there will be no difference in operative time after proficiency is achieved.
2. The HURWA system is expected to provide a bone cutting accuracy of approximately 1 mm, which should not be affected by the surgeon's experience or proficiency.
3. The HURWA system is anticipated to provide more accurate limb alignment adjustments than C-TKA, and this should not be affected by the surgeon's experience or proficiency.

## Materials and methods

### Patient selection

A retrospective study was conducted on a total of 119 cases of RA-TKA performed by three experienced arthroplasty surgeons after obtaining ethical clearance. The control group comprised the last 30 C-TKA cases performed by each surgeon, where qualifying imaging profiles were obtained prior to the commencement of the study.

Inclusion criteria: 18 years of age or older with robotic-assisted primary total knee arthroplasty for KOA.

Exclusion criteria: BMI greater than 40, traumatic knee osteoarthritis, previous fracture malunion of the femur or tibia, neurological or psychiatric dysfunction, osteotomy orthopedic treatment of the knee, severe systemic disease (such as severe diabetes or severe coronary artery disease), the use of the legacy constrained condylar knee (LCCK), or ligament repair due to inadequate medial or lateral collateral ligament function were excluded from the study. Additionally, participants who switched to C-TKA due to special circumstances were also excluded.

Based on the inclusion and exclusion criteria, a total of 106 cases were deemed eligible for the study. Of these, the first 30 cases per surgeon were included, resulting in a total of 90 cases being analyzed in the study.

**Fig. 1:**
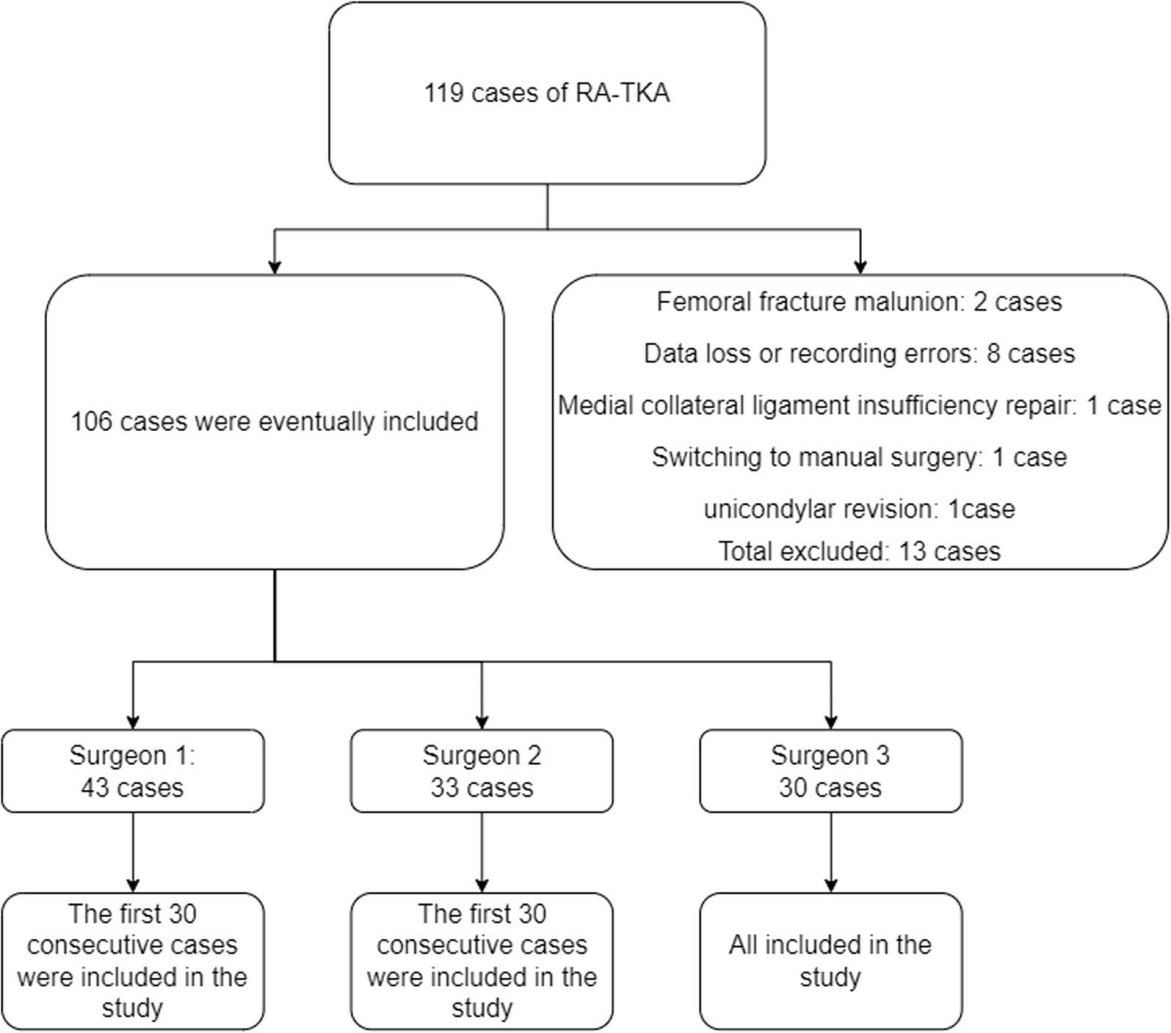
Flowchart of patient selection

Population characteristics were collected for each included patient, including age, sex (male = 0, female = 1), BMI, and surgical limb (left = 0, right = 1). In addition, the control and experimental groups will reflect the severity of osteoarthritis by measuring lower limb force lines. The measurements included the mechanical axis lateral distal femoral angle (mLDFA), medial proximal tibial angle (MPTA), and mechanical axis hip–knee–ankle angle (mHKA). ANOVA showed no significant differences in preoperative demographic characteristics or osteoarthritis severity between the test and control groups overall, indicating a consistent preoperative baseline (Fig. 1).

### Surgical technique

To ensure consistency in the study, all participating surgeons underwent comprehensive training in the HURWA system and performed bone simulation exercises prior to the first surgical operation. None of the surgeons had any prior experience with RA-TKA. During the operation, each surgeon was assisted by an attending physician and a resident doctor to ensure optimal surgical outcomes. Additionally, instrument nurses and circulating nurses were trained on the use of appropriate instruments prior to the operation to maintain consistency and accuracy throughout the study.

In both the test and control groups, a medial patellar approach was utilized without patellar replacement. Bone cement was used in all cases, and the tourniquet was released for hemostatic suturing after the cement had solidified. The operative time was measured from the start of the skin incision to the end of suturing (i.e., skin to skin) [6], and the time taken for positioning frame installation, bone registration, bone cutting, and total robot use were recorded separately as well. These measurements were conducted and recorded according to standardized protocols.

The initial 38 cases of RA-TKA utilized the Vanguard PS (Zimmer Biomet, Warsaw, IN, USA). However, due to national policy and copyright restrictions on implant parameters, starting from the 39th case, the femoral side of the Persona PS (Zimmer Biomet, Warsaw, IN, USA) and the tibial side and bearing of the NexGen (Zimmer Biomet, Warsaw, IN, USA) were employed. Detailed consultation with Zimmer confirmed that these two different types of implants can be used in conjunction with each other. For the control group, the entire NexGen set (Zimmer Biomet, Warsaw, Indiana, USA) was consistently used from start to finish, with no change to any other type of implant.

### Robot-assisted surgery system

The model of the robotic surgical system used in this experiment is HURWA-5800.This system is composed of several components, including a navigation console with an infrared camera tracking system, a trolley with a semiactive robotic arm and a pendulum saw, probes for joint contour calibration, a positioning frame for femur and tibia position capture, a fixed leg frame, and other orthopedic tools [9]. Before the surgery, the patient's CT scan data from hip to ankle with a continuous layer thickness of 0.6 mm were collected and imported into the HURWA local terminal and cloud platform for implant position planning and marking of the soft tissue safety line to ensure accurate and safe surgery.

The surgical procedure of the HURWA system consists of 3 steps [8]: installation of the positioning frame, bone registration, and bone cutting. The femoral condyle is first fixed to the leg brace's upright by a long screw to fix the knee position. The femoral positioning frame was installed inside the surgical incision in the nonbone cutting area of the anterior femoral cortex 1 cm from the trochlea. It is fixed to the femur using three locking screws distributed in a triangular pattern. The tibial positioning frame was installed in the mid-tibia outside the incision and fixed to the tibial crest using 2 locking screws.

The HURWA system has an advanced bone registration process that uses a probe with infrared reflection for topographic correction of the internal and external ankle, tibial plateau, and femoral condyle. Once the bone registration is completed, the surgeon can adjust the preoperative plan based on the individual patient's situation and then confirm the implant position and soft tissue safety line marking.

During the bone cutting process, the HURWA system provides real-time tracking of the robotic arm deviation distance to ensure accurate bone cutting. The system automatically powers off the robotic arm when it deviates from the intended bone cutting plane by more than 2 mm or near the soft tissue safety line to prevent injury. The surgeon can adjust the position of the arm and continue the operation once the deviation distance is within a safe range.

After the bone cutting is completed, the surgeon places a gap measurement pad and an implant trial mold in the joint space and performs flexion and extension gap and medial and lateral balance measurements through the femoral and tibial positioning frame. The surgeon can adjust the bone resection or perform soft tissue release if necessary to achieve a satisfactory gap and balance. Once the appropriate gap and balance are achieved, the final implant is inserted.

### Radiographic assessment

**Fig. 2:**
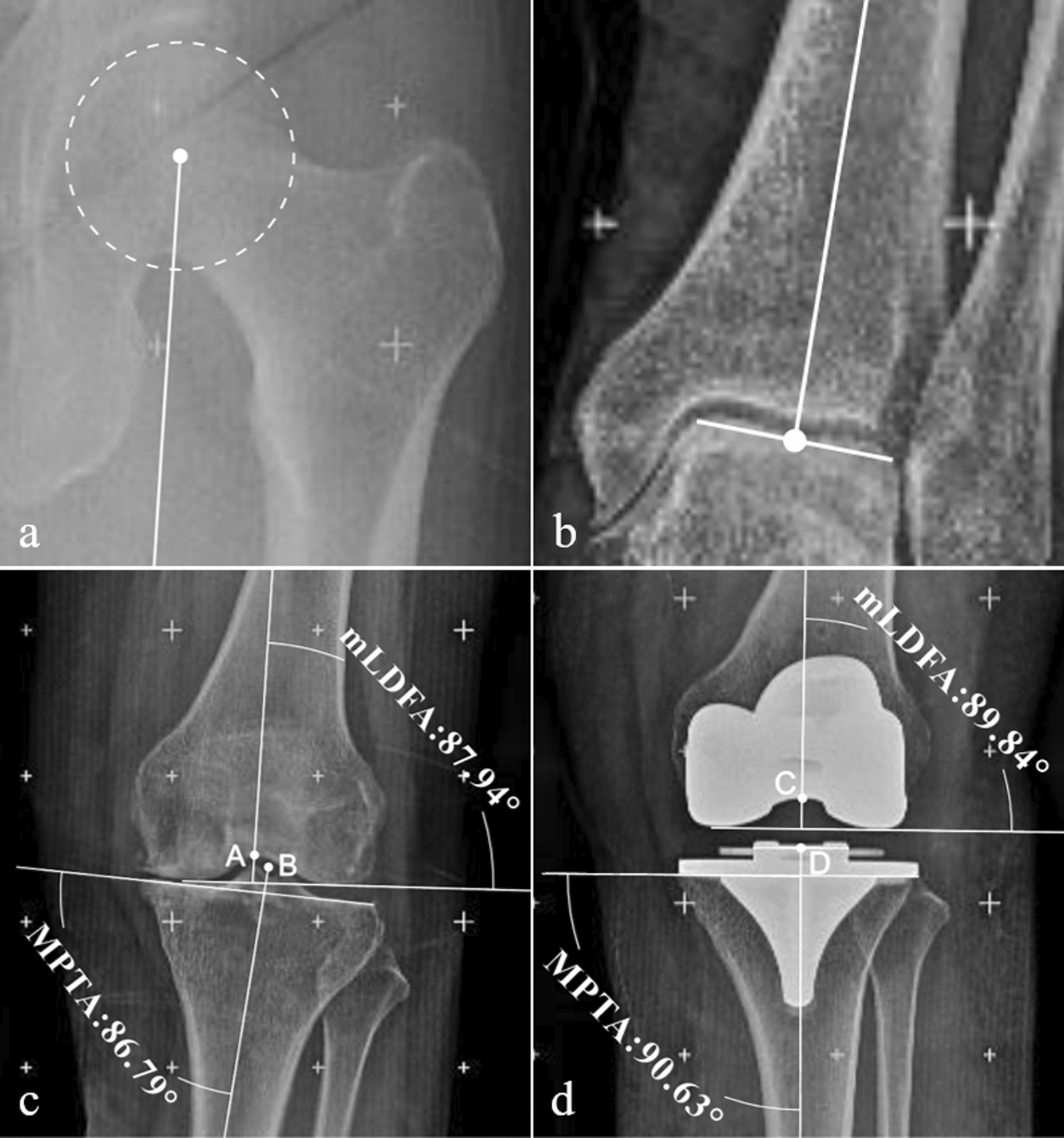
Diagram of the knee joint axis and angle. **a** The center of the femoral head was determined by the center of the MOSE circle. **b** The center of the ankle joint was the center of the articular surface of the talus. **c** Preoperative mLDFA and MPTA. Point A: Apex of the intercondylar. Point B: Center of interspinous groove. **d** Postoperative mLDFA and MPTA. Point C: Apex of the implant intercondylar. Point D: Center of the implant tibial plateau

On the preoperative and first postoperative day, patients were instructed to stand and walk on the ground and then undergo a full-leg radiograph. The standard full-leg radiograph position needed the patellar projection to be centered on both femoral condyles [10]. Postoperatively, due to the occlusion of the metallic shadow of the implant, the body markings of the patella were used to locate it. All images were acquired through the PACS system, and angular measurements were performed using CorelDRAW 2019 (Corel, Ottawa, Canada) software, with an accuracy of 2 decimal places. The center of the femoral head was determined using the center of the MOSE circle, and the femoral mechanical axis was defined as the straight line from the center of the femoral head to the apex of the intercondylar region [10] (Fig. 2). The measurements of the femoral and tibial mechanical axes were important in assessing the alignment of the lower limb after surgery. The mHKA was calculated as the angle formed between the mechanical axis of the femur and tibia, providing an overall assessment of limb alignment. The mLDFA was determined as the angle between the mechanical axis of the femur and a line drawn between the most distal medial and lateral femoral condyles. The MPTA was calculated as the angle formed between the mechanical axis of the tibia and a line drawn between the medial and lateral base of the tibial plateau. The joint line orientation (JLO) was then defined as the sum of mLDFA and MPTA [11].

This study focuses on the MA concept, which evaluates the accuracy of limb alignment based on two aspects: mHKA and JLO. The MA concept strives to achieve an mHKA angle of 180° and a JLO of 180° (mLDFA = MPTA = 90°). Postoperative mHKA angles that deviated from this target by more than 3° were recorded as abnormal values. In cases where patients had severe preoperative knee deformities, the MA target was not always feasible, and a residual partial varus or valgus was planned. There were 5 such cases with residual 2° or 3° valgus. In such cases, the postoperative limb alignment criteria were based on the actual planned mHKA angle ± 3°.

### Data collection and analysis

The accuracy of the bone cutting procedure was evaluated using a Vernier caliper with an accuracy of 0.1 mm [12]. Intraoperative measurements were taken on the bone fragments intercepted during the procedure, including the total bone cutting and the thickness of cartilage at the distal femur, posterior femoral condyle, and medial and lateral portions of the tibial plateau. Measurements were taken at a site consistent with the preoperatively planned reference point to ensure that the actual bone cutting at that point was consistent with the plan. The bone fragment was then cut along the sagittal plane using a sharp bone knife. Subsequently, Vernier calipers were employed to measure both the total thickness of the section and the cartilage thickness. The recorded data were averaged by two individuals and documented by a third person. The bone cutting error was calculated as the total bone cutting plus saw blade thickness minus cartilage thickness minus the preoperative planning value. The total bone cutting error for each case was calculated as the average of the absolute values of the bone cutting error at each site.

The performance of both bone cutting accuracy and operative time was evaluated using the CUSUM [13], which is a statistical quality control method used to monitor changes in the performance of a process over time. In this study, the learning curve of each surgeon was assessed using CUSUM, with the average bone cutting error and operative time for the first 30 cases of each surgeon set as the standardized target values. The difference between each subsequent data point and the standardized target was accumulated sequentially, and the CUSUM curve was plotted using the number of cases as the horizontal coordinate and the accumulated values as the vertical coordinate. The “inflection point” of the curve was identified as the transition point from the learning stage to the proficiency stage.

The data were analyzed using IBM SPSS 21 (IBM Corp., Armonk, NY, USA). Continuous variables were described using means and standard deviations (SDs), while categorical variables were reported as frequencies and percentages. To determine the difference between C-TKA and RA-TKA performed by the same surgeon, we used a two-sample heteroscedasticity t test. The differences between the three surgeons were analyzed using ANOVA. A statistically significant difference was considered when the p value was less than 0.05 (*P* < 0.05).

## Result

Demographic characteristics and preoperative mHKA analysis were performed in all cases of the 3 surgeons, and there were no statistically significant differences (Table 1).

**Table 1:** Demographic characteristics and preoperative baseline for the test and control groups

Demographic characteristics	surgeon 1		Surgeon 2		Surgeon 3		*P* value
	Test	Control	Test	Control	Test	Control	
Age	68.30 (8.31)	67.50 (7.99)	67.87 (8.13)	70.17 (7.71)	64.87 (7.54)	67.77 (8.51)	0.245
BMI	27.00 (3.26)	26.01 (2.78)	27.20 (3.85)	25.54 (3.19)	26.59 (3.77)	26.18 (4.12)	0.452
Gender (Male = 0, Female = 1)	0.77 (0.43)	0.93 (0.25)	0.87 (0.35)	0.93 (0.25)	0.90 (0.31)	0.93 (0.25)	0.262
Limb (Left = 0, Right = 1)	0.50 (0.51)	0.50 (0.51)	0.53 (0.51)	0.47 (0.51)	0.57 (0.50)	0.57 (0.50)	0.965
Pre mHKA	169.88 (9.01)	176.03 (8.16)	173.54 (10.07)	171.48 (8.99)	173.09 (9.41)	172.04 (7.82)	0.156
Pre mLDFA	89.13 (2.83)	87.75 (4.57)	87.91 (4.41)	88.53 (3.09)	87.1 (3.14)	88.91 (2.58)	0.245
Pre MPTA	85.74 (4.59)	86.67 (3.07)	85.57 (3.93)	84.30 (2.94)	84.64 (4.43)	85.11 (3.95)	0.214

### Time

Surgeon 1: The operative time was 111 ± 20.73 min in the first 30 cases, making a linear regression equation of *y* = −1.3041*x* + 131.21. The CUSUM curve reached a plateau in the 8th case, and the plateau continued until the 20th case when it started to decline.

Surgeon 2: The operative time in the first 30 cases was 113.43 ± 20.98 min, making a linear regression equation of *y* = −1.7542*x* + 140.62. The CUSUM curve reached the inflection point in the 16th case, after which it began to decline.

**Fig. 3:**
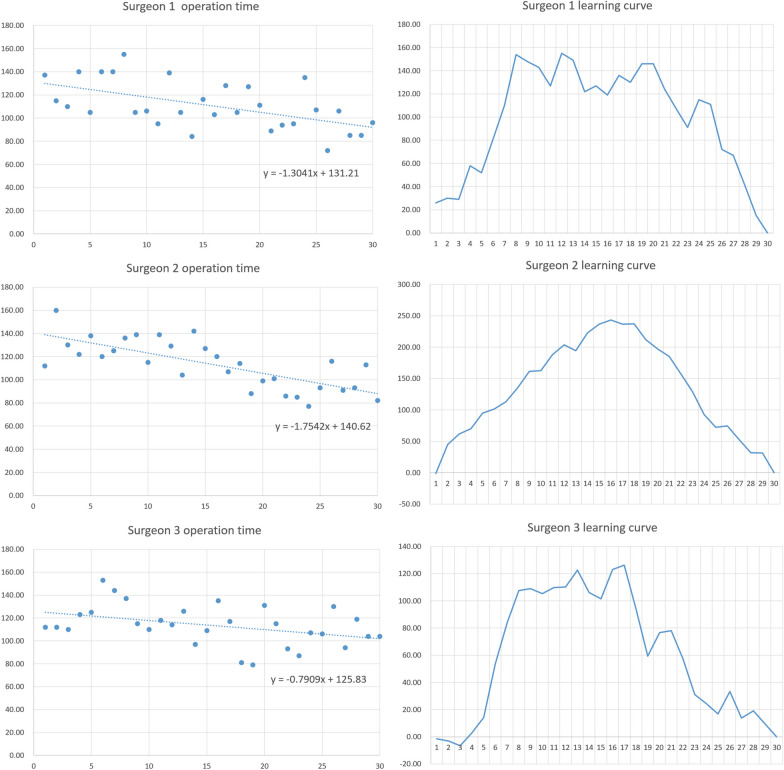
Operative time and CUSUM curve for 3 surgeons

Surgeon 3: The operative time was 113.57 ± 17.5 min in the first 30 cases, yielding a linear regression equation of *y* = −0.7909*x* + 125.83. The CUSUM curve reached a plateau in the 9th case, and the plateau continued until the 17th case when it started to decline (Fig. 3).

Surgeon 1, who had the longest career and the highest number of C-TKA procedures per year compared to Surgeon 2 and Surgeon 3, was able to complete the bone cutting operation in the shortest time (5.70 ± 2.58 min) and reached the inflection point of the learning curve first. Surgeon 2 and Surgeon 3, on the other hand, took longer to complete the bone cutting operation (8.36 ± 2.30 min and 10.15 ± 2.52 min, respectively) (Table 2).

**Table 2:** Operative time for 3 surgeons

Time (min)	Surgeon 1	Surgeon 2	Surgeon 3	*P* value
Total operative time (test)	111.00 (20.73)	113.43 (20.98)	113.57 (17.50)	0.853
Total operative time (control)	84.50 (15.92)	96.93 (23.31)	86.40 (14.36)	0.021
Last 10 cases total operative time (test)	96.40 (17.04)	93.7 (12.83)	105.9 (12.95)	0.158
Installation of the positioning frame	6.12 (2.34)	6.27 (2.57)	6.54 (2.94)	0.819
Bone registration	7.08 (2.01)	6.91 (2.20)	6.80 (2.43)	0.881
Bone cutting	5.70 (2.58)	8.36 (2.30)	10.15 (2.52)	< 0.001
Total robot time	18.9 (3.56)	21.54 (4.31)	23.49 (4.82)	< 0.001

All three surgeons showed a decreasing trend in the CUSUM curve after 20 cases, indicating that they were in the proficiency stage in the last 10 cases. From the overall 30 cases per person, there was no significant difference in the operating time of the three surgeons. There was also no significant difference in surgery time for the last 10 cases per surgeon (*p* = 0.158). Therefore, the previously proposed assumption 1 is valid, as the learning curve for the HURWA system is before 20 cases and the surgeon's operative time is not affected by their C-TKA experience.

### Bone cutting accuracy

**Fig. 4:**
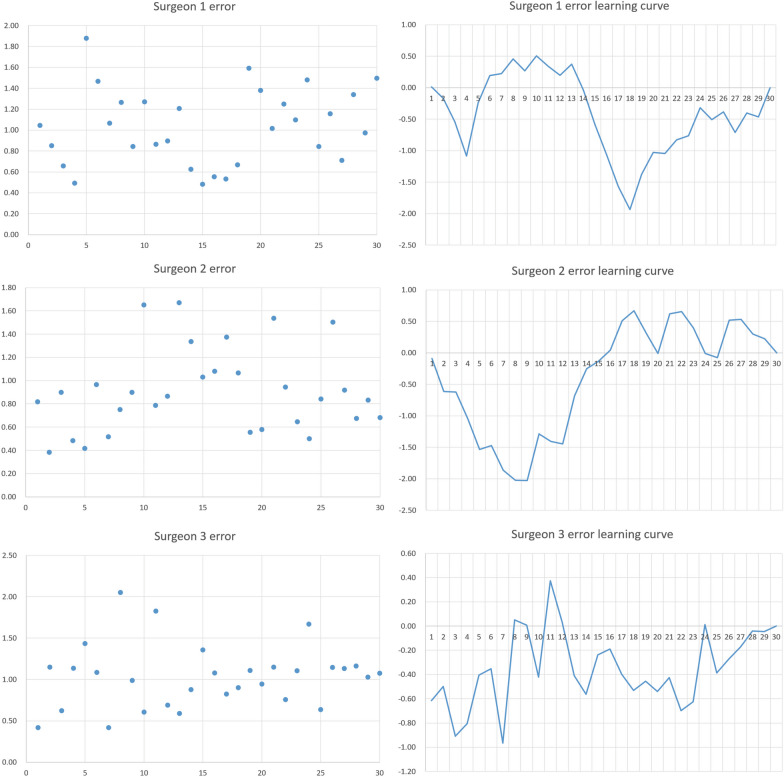
Bone cutting accuracy and CUSUM curve for 3 surgeons

The total bone cutting error for the three surgeons was 1.03 ± 0.36 mm, 0.91 ± 0.36 mm, and 1.03 ± 0.38 mm, respectively, with no significant difference among the three surgeons at each bone cutting site. The absence of an obvious inflection point or plateau in the CUSUM curve suggests that there is no learning curve for bone cutting accuracy (Fig. 4).

These findings imply that bone cutting accuracy is not affected by C-TKA experience or RA-TKA proficiency. The average error of 0.99 mm (SD 0.37 mm) was obtained by integrating the bone cutting errors of all three surgeons. Therefore, Assumption 2 is also valid (Table 3).

**Table 3:** Bone cutting error for 3 surgeons

Error (mm)	Surgeon 1	Surgeon 2	Surgeon 3	*P* value
Total error	1.03 (0.36)	0.91 (0.36)	1.03 (0.38)	0.319
Medial distal femur	0.98 (0.70)	0.64 (0.49)	1.09 (0.95)	0.056
Lateral distal femur	1.02 (0.83)	0.73 (0.72)	0.98 (0.71)	0.285
Medial posterior femoral condyle	1.03 (0.56)	0.80 (0.55)	0.91 (0.82)	0.398
Lateral posterior femoral condyle	0.90 (0.85)	1.04 (0.82)	0.87 (0.64)	0.667
Medial tibial plateau	1.20 (0.74)	1.02 (0.84)	1.38 (0.95)	0.282
Lateral tibial plateau	1.07 (0.74)	1.21 (0.83)	0.97 (0.80)	0.504

### Limb alignment

Since patients who underwent TKA usually have a full-leg radiograph taken on the first postoperative day, some patients were unable to obtain a standard full-leg radiograph due to residual flexion, internal and external rotation, or incisional pain. As a result, only 59 out of 90 cases were able to obtain a standard full-leg radiograph. Standard full-leg radiographs were not obtained in any of the 5 cases with preoperative planning for residual valgus.

The comparison of data from C-TKA and RA-TKA performed by the three surgeons revealed no significant difference in the postoperative lower limb mHKA angle, with mean values ranging from 177° to 183°. However, the mean and standard deviation of RA-TKA were smaller than those of the C-TKA group relative to the absolute value of 180° offset, indicating that RA-TKA has higher reproducibility and reduces the scatter of abnormal values. This could be due to the smaller standard deviation of the RA-TKA alignment error, resulting in a higher number of postoperative alignment errors falling within ± 3° than in the C-TKA group. There was also no significant difference in the alignment error between the three surgeons (Table 4).

**Table 4:** Force line and joint line accuracy for 3 surgeons

Force line accuracy	Surgeon 1	Surgeon 2	Surgeon 3	*P* value
Post mHKA				
Test	178.70 (2.21)	178.43 (1.93)	178.25 (2.05)	0.793
Control	177.8 (4.2)	177.12 (4.20)	178.29 (4.56)	0.575
*P* value	0.322	0.143	0.967	–
Post mHKA deviation value (°)				
Test	1.87 (1.59)	1.91 (1.41)	2.24 (1.45)	0.709
Control	3.42 (3.21)	3.97 (3.15)	4.15 (2.44)	0.606
*P* value	0.029	0.003	0.001	–
Number of cases within ± 3°/total (percentage)				
Test	16/20 (80%)	15/21 (71.4%)	14/18 (77.8%)	–
Control	17/30 (56.7%)	14/30 (46.7%)	11/30 (36.7%)	–
mLDFA				
Test	90.38 (1.68)	90.64 (1.47)	91.18 (1.74)	0.318
Control	90.84 (2.79)	90.85 (2.25)	90.89 (2.29)	0.997
*P* value	0.477	0.695	0.619	–
MPTA				
Test	89.34 (1.69)	88.85 (1.33)	89.47 (1.96)	0.467
Control	88.64 (1.50)	88.15 (2.65)	89.14 (2.68)	0.269
*P* value	0.141	0.225	0.634	–
JLO				
Test	179.72 (2.33)	179.49 (2.05)	180.64 (2.68)	0.286
Control	179.47 (2.55)	179.00 (2.86)	180.03 (3.04)	0.375
*P* value	0.724	0.483	0.467	–

For mLDFA, MPTA, and JLO, the means of the three surgeons were not significantly different between the C-TKA and RA-TKA groups, and the standard deviation of RA-TKA was smaller. This further demonstrated the high reproducibility of RA-TKA, as well as the smaller range of fluctuations. Therefore, Assumption 3 is also valid.

## Conclusion

In the first 30 consecutive cases of RA-TKA per surgeon, the total operative time did not significantly differ between the surgeons. There was a learning curve observed between 10 and 20 cases. The experienced surgeons consumed the least amount of time during the robot-assisted bone cutting phase. The operative bone cutting errors were in the range of approximately 1 mm, with no significant difference observed and no apparent learning curve. Limb alignment could be achieved in over 78% of cases, regardless of the surgeon and without a learning curve.

## Discussion

The field of joint surgery has seen significant advancements in recent years with the advent of surgical robots. These robots have been used to assist in a range of joint replacement procedures, such as THA, TKA, and UKA. Reports suggest that the use of robotic assistance in TKA can significantly reduce postoperative opioid use, improve limb alignment, and minimize surgical complications. These benefits have led to a rapid increase in the number of RA-TKA procedures being performed globally [14].

Dissatisfaction rates of up to 20% in C-TKA patients [15, 16] emphasize the need for improved surgical techniques. Poor implant positioning, limb alignment, and soft tissue imbalance are the main reasons for this high dissatisfaction rate [17]. RA-TKA can address these issues by providing precise surgical planning and intraoperative navigation, which leads to better postoperative outcomes. RA-TKA also helps to overcome the limitations of inexperienced surgeons, making it possible to achieve the MA concept, KA concept, or even FA concept. By adopting these concepts, RA-TKA can achieve excellent limb alignment and prosthesis positioning, leading to higher patient satisfaction rates [4]. The current accepted MA concept seeks to restore the patient's mHKA angle to 0 ± 3°. Commercially available MAKO, NAVIO, and ROSA robots have been shown to achieve postoperative mHKA angles with a mean ranging from 0.55° (SD 1.9°) to 1.2° (SD 1.1°) [7, 18, 19].

Recently, a new HURWA surgical robotic system received NMPA certification. Zheng et al. [9] conducted a study on 73 patients who underwent HURWA RA-TKA, and the results are promising. They were able to achieve a postoperative mHKA of 1.801 ± 1.608°, with a neutral alignment rate of 81.2%, which is consistent with the MA concept.

The learning curve is an important factor to consider when implementing a new surgical technique, as it reflects the number of cases a surgeon needs to perform to become proficient in the procedure. Previous studies have reported different learning curves for various robotic systems, ranging from 6 to 11 cases for the ROSA robot [20] to 7 cases for the image-based MAKO system [6]. The learning curve for the HURWA robot, an image-based semiautonomous robotic arm with an open platform, has not yet been reported.

It is known from the literature that the learning curve of other models of surgical robots is generally less than 20 cases [4], so to analyze the learning curve for RA-TKA with the HURWA robot, this study examined the first 30 consecutive cases performed by three surgeons with varying levels of experience. The primary objective was to determine whether the HURWA robot has a learning curve in terms of operative time, osteotomy accuracy, and limb alignment.

The results of this study showed that there was a learning curve for the use of the HURWA robot in RA-TKA, with experienced surgeons taking less time during the bone cutting phase. However, there was no significant difference in operative time, bone cutting accuracy, or limb alignment between the three surgeons, suggesting that robotic assistance could help overcome the experience barrier and enable more surgeons to perform TKA. Overall, these findings highlight the potential of robotic systems such as HURWA to improve surgical outcomes and expand access to joint replacement procedures.

The HURWA Surgical System, as an open platform, requires authorization from the implant company for intraoperative use and provision of the implant parameters. Currently, HURWA is authorized by Zimmer for the Vanguard PS complete implants, Persona PS for the femoral side, and NexGen for the tibial side. From the 39th RA-TKA onward, the state, aiming to reduce patient healthcare expenditures by centralizing the procurement of implants, excluded Vanguard PS from the list. Consequently, we changed the implant to the femoral side of Persona PS and the tibial side and bearing of NexGen. Preoperatively, we confirmed with Zimmer that these two implants could be paired. For the three implants mentioned above, different surgical instruments are used for positioning and bone cutting in C-TKA. However, for the HURWA surgical system, the trajectory of the robotic arm and the number of bone cutting planes remain the same regardless of the type of implants. Therefore, the use of different types of implants has minimal impact on the operative time of RA-TKA. In the control group, we used the entire NexGen implant throughout to avoid the effects of changing implant types and surgical instruments on the operating time.

From the results, it can be seen that surgeons can reach a stage of proficiency in operative time with 10–20 procedures without relying on traditional surgical experience. The operative time for the three operators was significantly shorter after 20 cases than before but was still longer than in C-TKA. This is a common phenomenon because RA-TKA involves processes such as mounting the positioning frame, bone registration, and robotic arm positioning and operation, which make the operation time longer. At the same time, the disadvantages of longer tourniquet time and longer incision exposure cannot be ignored. By continuously optimizing the operating procedure and gradually becoming more proficient, it is expected that the operating time of RA-TKA will be reduced to a level close to that of C-TKA. As the error control mechanism of the robotic arm and the safety line protection mechanism are independent of the operator, and the prosthesis position and lower limb alignment are highly reproducible both between and within operators and can be considered proficient from the beginning. This has important implications for the dissemination of TKA surgical techniques at the primary level.

It should be noted that by plotting a linear regression curve of the average bone cutting error in this study, it was found that the average error in RA-TKA increased by 0.0052 mm for each case, which may indicate a systematic error in the robot. As a result, it is recommended that the robot be recalibrated after a certain number of procedures.

This study has some limitations that need to be considered. First, the sample size is relatively small and further cases are needed to validate the HURWA system. Second, it was difficult to ensure that all patients received standard postoperative full-leg radiographs. Approximately 1/3 of the patients had knee flexion or rotation due to pain or other reasons, which may have affected the postoperative assessment of limb alignment. Additionally, it is important to note that the HURWA surgical system should not be limited to the MA concept of RA-TKA, and the surgical outcomes of the KA concept of individualized osteotomy should also be explored in subsequent studies. Finally, the measurement of bone cutting accuracy may have errors. Our aim is to validate the errors generated by the robot during the execution of the preoperative plan, so we want to choose the same points for the measurements as for the planning as much as possible. It is easier to find reference points on the femoral side. However, there is still no guarantee that the measurements at these points are exactly equal to the bone cutting accuracy. The points of the tibial side are relatively difficult to select due to the concave nature of the articular surface, and therefore, the accuracy of measurements on the tibial side may be slightly off compared to the femoral side. Lin et al. [21] reported that the HURWA system can achieve a bone cut error of 0.6 mm when operating in a prosthesis, but in real cases, the patient's bone and cartilage are more complex, and therefore, the level of error may be mildly elevated.

In conclusion, the HURWA system is a safe and precise system for total knee arthroplasty that can significantly reduce postoperative alignment abnormalities under the MA concept, with minimal reliance on the experience of the C-TKA procedure. However, additional long-term follow-up is necessary to validate its efficacy, and further studies are needed to explore potential additional benefits.
